# Protocol to identify mechanosensitive nuclear proteins using tunable actomyosin contractility and proximity biotinylation in mammalian cells

**DOI:** 10.1016/j.xpro.2025.104288

**Published:** 2025-12-24

**Authors:** Pei-Li Tseng, Weiwei Sun, Jiawei Li, Mark O. Collins, Kai S. Erdmann

**Affiliations:** 1 School of Biosciences, University of Sheffield, Sheffield S10 2TN, UK; 2 biOMICS Mass Spectrometry Facility, University of Sheffield, Sheffield S10 2TN, UK

**Keywords:** Biophysics, Cell biology, Molecular biology

## Abstract

Mechanical forces influence a range of cellular behaviors; however, how these forces are sensed and converted into biochemical changes remains incompletely understood. A key aspect of mechanotransduction is the regulation of subcellular protein localization. Here, we present a protocol describing the engineering of cell lines with tunable actomyosin contractility combined with a proximity biotinylation strategy confined to the nucleus followed by liquid chromatography-tandem mass spectrometry (LC-MS/MS) analysis. This approach allows the identification of proteins whose nuclear localization is controlled by changes of actomyosin contractility.

For complete details on the use and execution of this protocol, please refer to Tseng et al.^1^

## Highlights

• Protocol for the identification of proteins with mechanosensitive nuclear localization
• Generation of a cell line with tunable actomyosin contractility
• Quantification of nuclear proteome using proximity biotinylation and mass spectrometry
• Candidate validation by monitoring subcellular localization upon direct mechanical cues

## Abstract (editor highlights)

Publisher’s note: Undertaking any experimental protocol requires adherence to local institutional guidelines for laboratory safety and ethics.

## Graphical abstract

**Figure:**
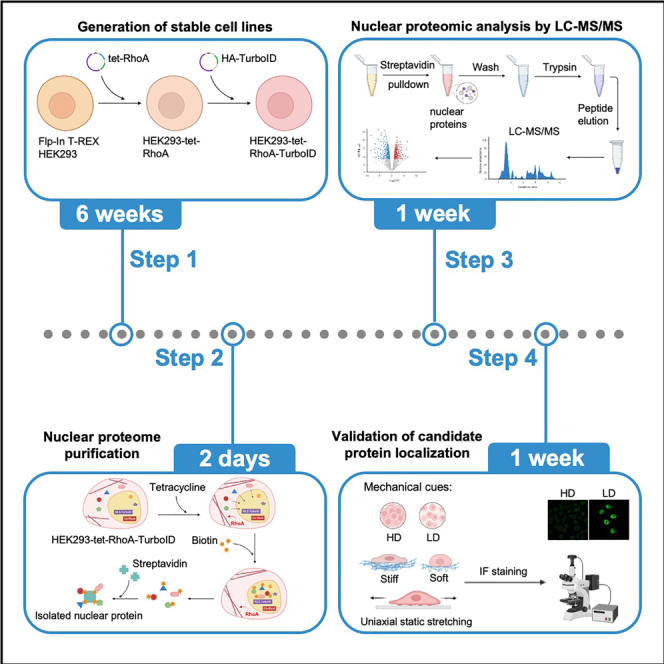

## Before you begin

Cells sense and respond to mechanical stimuli from their extracellular microenvironment through mechanotransduction, a process that converts physical forces into biochemical signals to regulate cell behavior, tissue homeostasis and disease progression.^2^^,^^3^^,^^4^ A central player of this process is the small GTPase RhoA, which is activated by external mechanical cues such as extracellular matrix stiffening and tension at adherens junctions.^5^ Upon activation, RhoA initiates a signaling cascade that drives actomyosin contractility and actin polymerization, dynamically reshaping the cytoskeleton to mediate cellular responses to force.^6^^,^^7^

Mechanical forces are transmitted to the nucleus via the Linker of Nucleoskeleton and Cytoskeleton (LINC) complex.^8^ This transmission leads to nuclear membrane deformation and modulates the architecture and function of nuclear pore complex,^9^ ultimately affecting nuclear transport, chromatin organization, and transcriptional regulation.^10^^,^^11^^,^^12^ These observations support the nucleus’s dual role as both a mechanical sensor and regulatory hub, highlighting the importance of identifying proteins with a mechanosensitive nuclear localization and to better understand their roles in mechanotransduction.

Physical methods to isolate intact nuclei for biochemical analysis have the potential to interfere with protein changes relevant to mechanotransduction. To circumvent this problem, we implemented nuclear-restricted biotinylation of cells in situ, allowing for the purification of nuclear proteins from cell lysates. Here we present a protocol to systematically identify proteins exhibiting mechanosensitive nuclear localization using a TurboID-based proximity labeling approach combined with label-free mass spectrometry. We utilize a HEK293-tet-RhoA-TurboID cell model, in which constitutively active RhoA is expressed under the control of a tetracycline-inducible promoter. Activation of RhoA mimics mechanical stimulation and triggers nuclear responses. The TurboID enzyme, fused to a nuclear localization signal, enables selective biotinylation of nuclear proteins in living cells, which can then be isolated and analyzed by mass spectrometry-based proteomics.

The protocol consists of three main parts: (1) generation of a cell model with tunable actomyosin contractility for screening proteins with a mechanosensitive nuclear localization, (2) quantitative proteomic analysis of biotinylated proteins by LC-MS/MS, and (3) validation of mechanosensitive subcellular localization of candidate proteins. We use YAP, a well-characterized mechanosensitive transcriptional regulator whose nuclear localization is modulated by mechanical stimuli,^13^^,^^14^ to validate our screen approach.

### Innovation

Approaches to modify actomyosin contractility in cells have been described before, however to our knowledge until now it has not been combined with proteomic approaches. Our protocol presents a systematic approach to identify proteins, which display a mechanosensitive nuclear localization, by combining a spatially restricted proximity biotinylation strategy allowing to selectively biotinylate the nuclear proteome with acute changes of actomyosin contractility using inducible constitutive RhoA expression. This is followed by a quantitative LC-MS/MS analysis. This provides a unique and scalable strategy to uncover nuclear proteomic changes driven by changes in actomyosin contractility allowing the systematic identification of previously unrecognized nuclear mediators of mechanical signaling.

### Construction of pcDNA5/FRT/TO-RhoA-Q63L

**Timing: 1 week**

To identify proteins that change nuclear abundance in response to mechanical stimuli, we employ an approach that mimics mechanosensitive signaling initiated by RhoA. In this protocol, we engineer a plasmid encoding an EGFP-tagged, constitutively active RhoA variant (RhoA-Q63L) under a tetracycline inducible promoter. Using standard cloning techniques, we isolate EGFP-RhoA-Q63L from donor plasmid pcDNA3-EGFP-RhoA-Q63L and subclone it into the recipient plasmid pcDNA5/FRT/TO to generate pcDNA5/FRT/TO-RhoA-Q63L.

***Note:*** The construction of pcDNA5/FRT/TO-RhoA-Q63L is performed using standard molecular cloning techniques including PCR amplification of EGFR-RhoA-Q63L from donor plasmid, followed by restriction enzyme digestion and ligation into recipient plasmid pcDNA5/FRT/TO. The choice of restriction enzymes is based on the presence of unique sites of pcDNA5/FRT/TO and their absence within EGFR-RhoA-Q63L insert to ensure correct orientation and integrity of the cloned sequence. The final construct was verified by Sanger DNA sequencing.

### Construction of pcDNA3.1-Puro-3xHA-TurboID-NLS

**Timing: 1 week**

**Figure 1:**
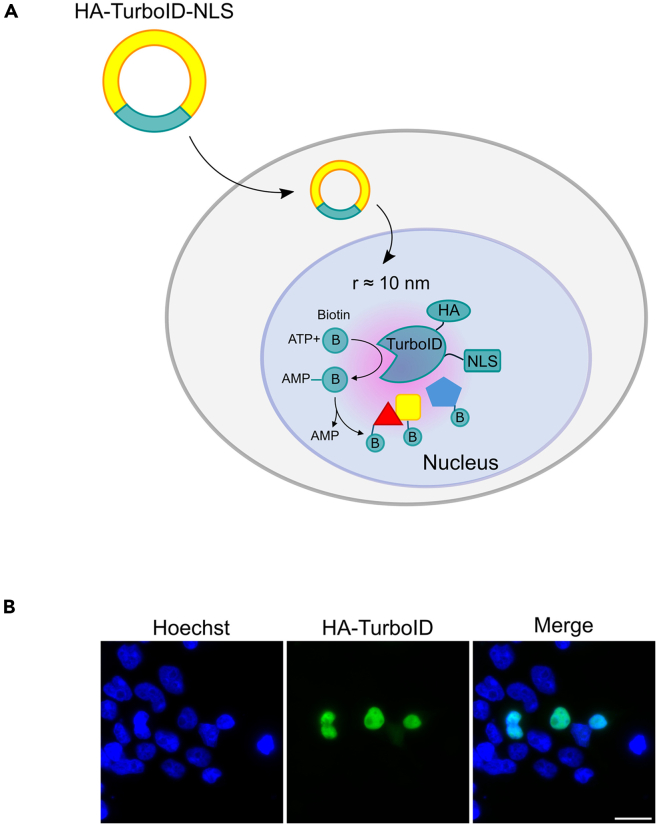
Nuclear localization of TurboID-NLS (A) Schematic representation of 3xHA-TurboID-NLS mediated proximity biotinylation. (B) Immunofluorescence staining confirms nuclear localization of 3xHA-TurboID-NLS in transiently transfected HEK293 cells. Bar represents 50 μm.

TurboID is an engineered biotin ligase variant with enhanced biotinylation kinetics, capable of rapid labelling of proximal proteins within a ∼10 nm radius.^15^^,^^16^ To biotinylate selective nuclear proteins, we utilize a plasmid coding for an HA-tagged TurboID including three nuclear localization signals (NLS) for nuclear-restricted TurboID expression (Figure 1A), which can be verified by immunofluorescence staining using anti-HA antibody (Figure 1B). Using standard cloning techniques, we isolate 3xHA-TurboID-NLS from the donor plasmid 3xHA-TurboID-NLS_pCDNA3 and insert into the recipient vector pCDNA3.1-PuroR to create recombinant plasmid pcDNA3.1-Puro-3xHA-TurboID-NLS. This construct enables nuclear specific protein labelling and puromycin selection of transfected cells.

***Note:*** The 3xHA-TurboID-NLS_pCDNA3 (Addgene_107171) contains a *Neo/KanR* resistance cassette for selection with geneticin. However, our kill curve test shows that HEK293-tet-RhoA cells are weakly sensitive to geneticin, even after 5 days of culture at concentration ranging from 100 to 800 μg/ml, which hinders the selection of stable clones. Thus, we subcloned 3xHA-TurboID-NLS into the pCDNA3.1-PuroR to generate pcDNA3.1-Puro-3xHA-TurboID-NLS. If your cells are sensitive to geneticin, you can skip this recloning step.

## Key resources table

**Table:** 

REAGENT or RESOURCE	SOURCE	IDENTIFIER
**Antibodies**		

Mouse monoclonal anti-YAP, antibody dilution: 1/200	Santa Cruz	#sc-101199; RRID: AB_1131430
Rabbit polyclonal anti-GFP, antibody dilution: 1/2,000	Invitrogen	#A-11122; RRID: AB_221569
Mouse monoclonal anti-nucleolin, antibody dilution: 1/300	Santa Cruz	#sc-8031; RRID: AB_670271
Mouse monoclonal anti-β-tubulin, antibody dilution: 1/5,000	Sigma-Aldrich	#T8328; RRID: AB_1844090
Mouse monoclonal anti-γ-adaptin, antibody dilution: 1/10,000	BD Biosciences	#610385; RRID: AB_397768
Rabbit monoclonal anti-HA tag, antibody dilution: 1/1,000	Cell Signaling	#3724; RRID: AB_1549585
Mouse monoclonal anti-PTBP1, antibody dilution: 1/300	Thermo Fisher Scientific	#32-4800; RRID: AB_2533082
Alexa Fluor 680 Streptavidin Conjugate, antibody dilution: 1/5,000	Thermo Fisher Scientific	#S32358
Donkey anti-mouse IgG (H+L) Alexa Fluor 594, antibody dilution: 1/500	Thermo Fisher Scientific	#A-11005; RRID: AB_2534073
Goat anti-mouse IgG (H+L) Alexa Fluor 488, antibody dilution: 1/500	Thermo Fisher Scientific	#A-11001; RRID: AB_2534069
DyLight 800 4X PEG conjugate anti- mouse IgG (H+L), antibody dilution: 1/5,000	Thermo Fisher Scientific	#SA5-35521; RRID: AB_2556774

**Experimental models: Cell lines**		

Flp-In T-REX HEK293	Thermo Fisher Scientific	RRID: CVCL_U427
MCF10A	ATCC	#CRL-10317; RRID: CVCL_0598
NIH3T3	ATCC	#CRL-1658; RRID: CVCL_0594
HEK293-tet-RhoA-TurboID	This study	N/A

**Chemicals, peptides, and recombinant proteins**		

DMEM-GlutaMax	Gibco	#10569010
Blasticidin S HCl	Gibco	#R21001
Zeocin Selection Reagent	Gibco	#R25001
Hygromycin B	Gibco	#10687010
Puromycin dihydrochloride	Santa Cruz	#sc-108071
Tetracycline hydrochloride	Sigma-Aldrich	#T7660
Biotin	Sigma-Aldrich	#B4501
Collagen I, rat tail	Gibco	#A1048301
Hoechst 33342, trihydrochloride	Thermo Fisher Scientific	#H3570
Poly-L-Lysine solution	Sigma-Aldrich	#P8920
Formaldehyde solution	Sigma-Aldrich	#252549
Triton X-100	Sigma-Aldrich	#X100
TWEEN 20	Sigma-Aldrich	#P6585
ML-7, hydrochloride	Sigma-Aldrich	#475880
ProLong Gold Antifade Mountant	Invitrogen	#P36930
Dimethyl Sulfoxide Hybri-MaxTM Sterile-Filtered BioReagent (DMSO)	Sigma-Aldrich	#D2650-100ML
Pierce Streptavidin Agarose	Thermo Fisher Scientific	#20349
Lipofectamine 2000	Thermo Fisher Scientific	#11668019
cOmplete, EDTA-free Protease Inhibitor Cocktail	Sigma-Aldrich	#11873580001
Ammonium bicarbonate	Thermo Fisher Scientific	#393212500
TCEP (Tris(2-carboxyethyl)phosphine)	Sigma-Aldrich	#C4706
IAA (Iodoacetamide)	Sigma-Aldrich	#I1149
TFA (Trifluoroacetic acid)	Sigma-Aldrich	#T6508
ACN (Acetonitrile)	Sigma-Aldrich	#34851
Formic Acid	Honeywell Chemicals	#9431850ML
Water, for HPLC-MS, Fisher Chemical	Fisher Scientific	#10777404

**Deposited data**		

Proteomics Data		PRIDE: PXD046157

**Recombinant DNA**		

pcDNA5/FRT/TO	Thermo Fisher Scientific	#V652020
pOG44	Thermo Fisher Scientific	#V600520
pcDNA3-EGFP-RhoA-Q63L	Addgene	RRID: Addgene_12968
3xHA-TurboID-NLS_pCDNA3	Addgene	RRID: Addgene_107171
pcDNA3.1 (+)-myc-PuroR	Lab of Dr. Kai Erdmann	N/A

**Software and algorithms**		

MaxQuant 1.6.2.6	MaxQuant	RRID:SCR_014485
Perseus 1.5.6.0	MaxQuant	RRID:SCR_015753
Fiji (ImageJ)	Schindelin et al.^17^	RRID:SCR_002285
ImageStudioLite	LI-COR Biosciences	N/A
GraphPad Prism 9.5.1	Prism	RRID:SCR_002798
Biorender	Biorender	RRID:SCR_018361

**Other**		

Scienceware cloning discs	Sigma-Aldrich	Discontinued
Coverslips 18 mm	SLS	#MIC3342
Pierce C18 stage tips	Thermo Fisher Scientific	#87782
Wizard^R^ Minicolumn	Promega	#A7211
Orbitrap Elite Hybrid Mass Spectrometer	Thermo Fisher Scientific	
Petrisoft 35 (0.2 kPa)	Matrigen	#PS35-COL-0.2
Petrosoft 35 (25 kPa)	Matrigen	#PS35-COL-25
Petrisoft 35 (12 kPa, embedded with 0.2 μm red spheres)	Matrigen	#PS35-COL-12-ST0.2R
Odyssey Sa Infrared Imaging System	LI-COR	N/A
Olympus Fluoview 1000 Confocal Microscopy	Olympus	N/A
Vacuum concentrator	SpeedVac, Eppendorf	N/A
ZEISS Celldiscoverer 7	ZEISS	N/A
Manual Stretch Device (4 cm^2^ uniaxial)	STREX	#ST-0040
PDMS uniaxial stretch chamber (2 cm^2^ wells)	STREX	#SC-0044

## Materials and equipment

**Table:** DMEM medium for Flp-IN TREX HEK293 (500 ml)

Reagent	Final concentration	Amount
DMEM-GlutaMax	N/A	443.75 ml
Fetal bovine serum (FBS)	10 %	50 ml
100x Penicillin/Streptomycin	1%	5 ml
Zeocin (100 mg/ml)	100 μg/ml	500 μl
Blasticidin S HCl (10 mg/ml)	15 μg/ml	750 μl

Store at 4°C for up to six months.

**Table:** DMEM medium for HEK293-tet-RhoA-TurboID (500 ml)

Reagent	Final concentration	Amount
DMEM-GlutaMax	N/A	442.25 ml
Fetal bovine serum (FBS)	10 %	50 ml
100x Penicillin/Streptomycin	1%	5 ml
Blasticidin S HCl (10 mg/ml)	15 μg/ml	750 μl
Hygromycin B (50 mg/ml)	200 μg/ml	2 ml

Store at 4°C for up to six months.

**Table:** 1000x Tetracycline stock solution (1 ml)

Reagent	Final concentration	Amount
Tetracycline	1 mg/ml	1 mg
70% Ethanol	N/A	1 ml

Aliquot and store at −20°C for up to 1 month.

**Table:** 100x Biotin solution (2 ml)

Reagent	Final concentration	Amount
Biotin	50 mM	24.43 mg
DMSO	N/A	2 ml

Store at −20°C for up to six months in single-use aliquots to avoid repeated freeze-thaw cycles.

**Table:** RIPA lysis buffer (500 ml)

Reagent	Final concentration	Amount
Tris-HCl, pH 7.5 (1 M)	50 mM	25 ml
NaCl (1 M)	150 mM	75 ml
10% SDS	0.1%	5 ml
Sodium deoxycholate	0.5%	2.5 g
EDTA (500 mM)	1 mM	1 ml
NP-40	1 %	5 ml
ddH_2_O	N/A	389 ml
Total	N/A	500 ml

Store at 4°C for up to six months.

**Table:** Lysis buffer (10 ml)

Reagent	Final concentration	Amount
RIPA lysis buffer	N/A	9.6 ml
cOmplete, EDTA-free Protease Inhibitor (25x)	1x	0.4 ml
Total	N/A	10 ml

Prepare freshly before use and keep on ice throughout the procedure.

**Table:** 4x Laemmli buffer (50 ml)

Reagent	Final concentration	Amount
Tris-HCl, pH 6.8 (1 M)	250 mM	12.5 ml
Glycerol	40%	20 ml
SDS	8%	4 g
β-mercaptoethanol	20%	10 ml
Bromophenol blue	1%	0.5 g
ddH_2_O	N/A	7.5 ml
Total	N/A	50 ml

Aliquot and store at −20°C for up to 12 months.

**Table:** PFA fixation buffer (10 ml)

Reagent	Final concentration	Amount
1xPBS (pH 7.4)	N/A	8.9 ml
Formaldehyde solution (37%)	4%	1.1 ml
Total	N/A	10 ml

Store at −20°C in single-use aliquots to avoid repeated freeze-thaw cycles.

**Table:** Permeabilization buffer (50 ml)

Reagent	Final concentration	Amount
1xPBS (pH 7.4)	N/A	49.95 ml
Triton X-100	0.1 %	0.05 ml
Total	N/A	50 ml

Store at 4°C for up to one week.

**Table:** Blocking and antibody incubation buffer (50 ml)

Reagent	Final concentration	Amount
1xPBS (pH 7.4)	N/A	49.745 ml
FBS	0.5 %	0.25 ml
TWEEN 20	0.01 %	0.005 ml
Total	N/A	50 ml

Stored at 4°C for up to one week.

## Step-by-step method details

### Generation of a cell model with tunable actomyosin contractility

**Timing: 6 weeks**

This section describes details of generating a stable HEK293-tet-RhoA-TurboID cell line by sequentially introducing two plasmids described above in the before you begin section, pcDNA5/FRT/TO-RhoA-Q63L and pcDNA3.1-Puro-3xHA-TurboID-NLS, into a host cell line Flp-IN TREX HEK293. The first plasmid can integrate into the host cell genome at a FRT integration site and express constitutively active RhoA variant (RhoA-Q63L) under the control of a tetracycline inducible promoter, while the second plasmid expresses TurboID biotin ligase for proximity labelling of the nuclear proteome.

***Note:*** Before starting to generate the above cell line, a kill curve experiment to determine the optimal concentration of the selecting drug for the establishment of stable clones should be performed. We test a range of hygromycin concentrations (100 to 700 μg/ml, in 50 μg/ml increments) and puromycin concentrations (1 to 10 μg/ml, in 0.5 μg/ml increments). The optimal concentrations are determined to be 200 μg/ml hygromycin and 1.5 μg/ml puromycin for selecting HEK293-tet-RhoA and HEK293-tet-RhoA-TurboID stable clones respectively, as these are the lowest concentrations that kill all non-resistant cells within 5 days.

1. Generation of HEK293-tet-RhoA cell line.
  a. Seed 4x10^5^ Flp-IN TREX HEK293 cells in a 35mm culture dish, when cells have reached a confluency of about 80% (after ca. 20 h) cells are ready for transfection.
  b. Prepare a mixture of DNA containing 9:1 ratio (w/w) of pOG44: pcDNA5/FRT/TO-RhoA-Q63L.
  c. Transfect DNA mixture into Flp-IN TREX HEK293 by Lipofectamine 2000 according to manufacturer’s instruction.
  d. After 24 h of transfection, replace the medium with fresh complete medium containing 15 μg/ml blasticidin. Continue to culture the cells for another 24 h.
    **CRITICAL:** Since the *lacZeo* gene in Flp-IN TREX HEK293 was interrupted by the integration of pcDNA5/FRT/TO-RhoA-Q63L into the FRT site, zeocin must be removed from the culture medium after transfection because the transfectants will become sensitive to zeocin.
  e. Split cells (1:10) in complete medium containing 15 μg/ml blasticidin into a new 6-well culture plate. Incubate the cells for 24 h to allow cells to attach.
  f. Start the selection of successful transfectants by replacing the medium with selection medium supplemented with 15 μg/ml blasticidin and 200 μg/ml hygromycin.
  g. Refresh the selection medium every 2–3 days until colonies appear under the observation with an inverted microscope. Pool all colonies by detaching the cells with trypsin and replating, which is expected to result in an isogenic population given the defined integration locus for the plasmid.
    **Pause point:** Cells can be frozen in the selection medium containing 10% DMSO and stored in liquid nitrogen.
    **CRITICAL:** It is important to validate the generated stable HEK293-tet-RhoA cell line before introducing the second plasmid. For this, we assessed EGFP expression by live fluorescence microscopy after 24 h treatment with 0.5 μg/ml tetracycline. We expected to observe isogenic EGFP expression across cell population, confirming the stable and inducible EGFP-RhoA-Q63L expression.
2. Generation of stable HEK293-tet-RhoA-TurboID cell line.
  ***Note:*** This part describes the further modification of the above cell line by introducing a nuclear localized Turbo protein biotin ligase allowing selective biotinylation of nuclear proteins.
  a. Seed 3x10^5^ HEK293-tet-RhoA in a 35 mm petri dish for overnight. The cells are ready for transfection when reaching about 80% of confluency.
  b. Perform transfection of 1 μg of linearized pcDNA3.1-Puro-3xHA-TurboID-NLS plasmid to cells using Lipofectamine™ 2000 according to manufacturer’s instruction.
  c. 24 h after transfection, replate and culture cells in a new 10 cm petri dish with complete medium for 24 h.
  d. By the time cells should reach around 30%–50% confluency. Start selecting puromycin-resistant transfectants with selection medium containing 1.5 μg/ml puromycin. Change selection medium every 2∼3 days until single colonies appear.
3. Isolate single cell colonies using cloning discs.
  ***Note:*** All the procedure must be done under sterile conditions. Select colonies that are of average size, approximately 1–2 mm in diameter. The Scienceware cloning disc (Z374431, Sigma-Aldrich) used in this protocol is now discontinued. However, alternative methods like using cloning cylinders (C7983, Sigma-Aldrich) can be considered to harvest the cells.
  a. Examine the cells with an inverted microscope to locate single colonies that are ready for isolation. Draw a circle around the selected colonies on the bottom of the dish with a marker pen.
  b. Aspirate culture medium and rinse the cells twice with prewarmed 1x PBS to remove floating cells. Leave a thin layer of PBS in the culture dish to avoid cells from drying.
  c. Use sterile forceps to place cloning disc over the selected colony, press gently on the cloning disc and carefully scrape the cell colony off with the disc.
  d. Transfer the disc to 24-well plate, with the disc side containing cell colony facing down.
  e. Continue to culture the cells in selection medium for two days before removing the disc.
    ***Note:*** It is time to remove the cloning disc when the cells have proliferated so they expanded out of the disc area.
  f. When cells reach around 70% confluency, replate the cells to a suitable culture vessel for further expansion.
    **Pause point:** Cells can be frozen in the selection medium containing 10% DMSO and stored in liquid nitrogen.

### Characterization of the stable HEK293-tet-RhoA-TurboID cell line

**Timing: 3 days**

**Figure 2:**
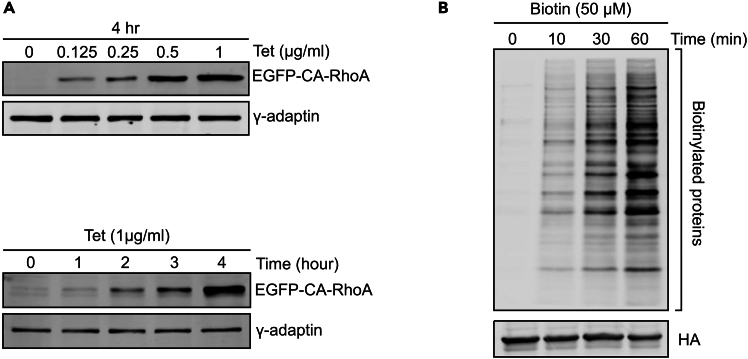
Inducible EGF-CA-RhoA and TurboID mediated biotinylation in HEK293-tet-RhoA-TurboID (A) EGFP-CA-RhoA expression in HEK293-tet-RhoA-TurboID can be controlled by tetracycline in a dose dependent (upper panel) and time dependent (lower panel) manner. γ-adaptin is used as loading control. (B) TurboID-catalyzed protein biotinylation in the presence of 50 μM Biotin for indicated time points. The biotinylated proteins were detected by streptavidin blotting. HA is used as loading control.

To validate the suitability of the selected clones for the intended screen, tetracycline dependent EGFP-CA-RhoA expression and protein target biotinylation should be confirmed in each stable HEK293-tet-RhoA-TurboID cell line generated (Figure 2).

4. EGFP-CA-RhoA expression analysis.
  a. Seed 5.5x10^5^ HEK293-tet-RhoA-TurboID cells in a 35 mm culture dish for 24 h to achieve 90% confluency.
  b. Incubate cells with varying doses of tetracycline and for different durations at 37°C.
    ***Note:*** To determine the optimal tetracycline treatment, we test a range of tetracycline concentrations (0.125∼1 μg/ml) for 4 h and 1 μg/ml tetracycline for various durations (1∼4 h) (Figure 2A). Based on these results, we select 1 μg/ml tetracycline for 2 h as standard treatment for subsequent experiments.
  c. Wash cells twice with ice-cold PBS. Prepare cell lysate using ice-cold lysis buffer following standard cell lysis procedure.
  d. Load 20 μg protein onto a 10% polyacrylamide gel and detect EGFP-CA-RhoA expression by western-blotting using EGFP antibody.
5. Protein biotinylation analysis.
  a. Culture HEK293-tet-RhoA-TurboID as Step 4a.
  b. Incubate cells with 50 μM biotin for varying time periods from 10–60 min at 37°C.
  c. Aspirate culture medium and wash the cells five times with ice-cold PBS to remove residual biotin.
  d. Prepare cell lysate as described in Step 4c.
  e. Load 20 μg protein onto a 10% polyacrylamide gel and detect biotinylated proteins by western blotting using Alexa Fluor™ 680 Streptavidin Conjugate.

### Functional validation of the stable HEK293-tet-RhoA-TurboID cell line

**Timing: 2 days**

**Figure 3:**
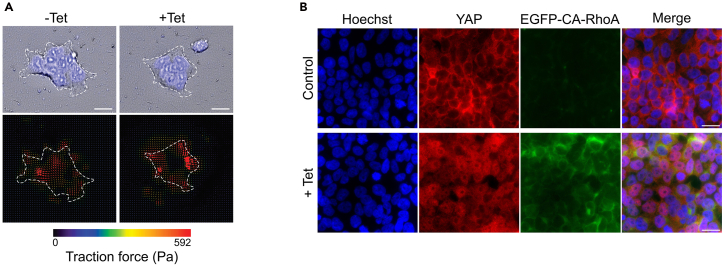
Tetracycline induced EGFP-CA-RhoA expression enhances actomyosin contractility and YAP nuclear accumulation (A) Detection of traction force in HEK293-tet-RhoA before and after two hours of tetracycline treatment. Cells were visualized by staining of nuclei. Red arrows indicate the force direction. Bar represents 20 μm. Data reprinted with permission from Tseng et al.^1^ (B) Immunofluorescence imaging shows the tetracycline-induced EGFP-CA-RhoA expression and subsequent YAP nuclear accumulation. Bar represents 20 μm.

The stable HEK293-tet-RhoA-TurboID cell line enables tetracycline-inducible expression of CA-RhoA, which is expected to enhance actomyosin contractility and promote nuclear accumulation of YAP. To confirm this is in the established HEK293-tet-RhoA-TurboID cell lines, we measure changes in cellular contractile forces and subcellular localization of YAP upon tetracycline induction (Figure 3).

6. Assess tetracycline induced cellular contractility.
  a. Plate 1x10^5^ HEK293-tet-RhoA-TurboID on 35 mm collagen-coated plates (stiffness: 12 kPa) embedded with 0.2 μm red fluorescent beads.
  b. Incubate for 24 h under standard condition.
  c. Replace medium with fresh medium containing 0.05 μg/ml Hoechst for nucleus counterstaining.
  d. Transfer the plate to an incubation chamber (37°C, 5% CO_2_) on the microscope stage.
  e. Acquire baseline images of the cell clusters and beads using a Cell Discoverer 7 microscope (Zeiss, Germany).
  f. Treat cells with 1 μg/ml tetracycline and acquire follow-up images after 2 h.
    ***Note:*** Concentration and time of tetracycline treatment will depend on the concentration and time-dependent EGFP-RhoA expression levels established above.
  g. Quantify bead displacements using particle image velocimetry (PIV). Calculate traction force via Fourier transform traction cytometry (FTTC) implemented in a modified ImageJ plugin incorporating slice alignment, PIV, and FTTC function.^18^^,^^19^ Total cellular traction force is calculated by integrating traction stresses over the cell area.
    **CRITICAL:** To avoid unreliable traction measurements, it is important to ensure that cells are well spread and adhere firmly to the collagen-coated substrate before imaging. Maintain focus stability and consistent imaging conditions throughout image acquisition. Minimize phototoxicity and photobleaching by optimizing exposure time. Use identical microscope and acquisition settings for baseline and post-tetracycline treatment images to ensure accurate displacement quantification.
7. Analyze subcellular localization of YAP by immunofluorescence imaging.
  a. Put sterile coverslip in 12-well and add 1 ml of Poly-L-Lysine (1:10 dilution in sterile water) to each well, incubate for 30 min at RT.
  b. Remove Poly-L-Lysine solution by aspiration, then allow the coverslips to dry completely.
  c. Immediately before seeding, rinse the coverslips three times with 1xPBS.
  d. Seed 5.5x10^5^ cells on Poly-L-Lysin coated coverslips and culture in puromycin-free medium for 24 h. The cells should reach 90% confluency before tetracycline treatment.
  e. Treat cells with 1 μg/ml tetracycline or 0.1 μl 70% Ethanol as vehicle control for 2 h at 37°C.
    ***Note:*** Concentration and time of tetracycline treatment will depend on the concentration and time dependent EGFP-RhoA expression levels established above.
  f. Wash the cells three times with 1x PBS, then fix the cells with PFA fixation buffer (500 μl/well) for 10 min at RT.
  g. Wash the cells three times with 1x PBS, then permeabilize cells with permeabilization buffer (1 ml/well) for 15 min at RT.
  h. Wash the cells three times with 1x PBS. Block with blocking buffer (1 ml/well) for 1 h at RT.
  i. Incubate with YAP primary antibody for 2 h at RT.
  j. Wash the cells three times with 1x PBST.
  k. Incubate the cells with secondary antibody and Hoechst for 1 h in the dark.
  l. Wash the cells three times with 1x PBST.
  m. Mount the coverslips onto clean glass slides with Prolong gold anti-fade mounting medium (3 μl/each slide).
    ***Note:*** After mounting, allow the medium to cure at least 24 h in the dark before imaging. Store the slides at 4°C in the dark for up to 1 week.
  n. Analyze the subcellular localization of YAP and EGFP-CA-RhoA expression using fluorescence microscope.

### Isolation of nuclear proteins by streptavidin pull-down

**Timing: 2 days**

8. Isolate nuclear proteins.
  a. Seed 6.5x10^6^ HEK293-tet-RhoA-TurboID in 100 mm dish for 24 hr to reach 90% confluency.
  b. Treat cells with 1 μg/ml tetracycline or 1 μl 70% Ethanol as vehicle control for 2 h at 37°C to induce EGFP-CA-RhoA expression.
  c. Add 500 μM biotin to the culture medium and incubate for 20 min at 37°C.
  d. Aspirate culture medium. Wash cells five times with ice-cold PBS to remove residual biotin.
  e. Lyse cells in ice-cold RIPA buffer following standard cell lysis procedure.
  f. Prepare streptavidin agarose beads for pulldown by aliquoting 100 μl of beads and washing them three times with ice-cold RIPA buffer by centrifugation at 1,800 x *g* for 2 min at 4°C.

***Note:*** Keep the streptavidin agarose beads on ice throughout the procedure to minimize protein degradation.

9. Remove the supernatant and add the cell lysate (as prepared above) containing about 3000 μg protein to the beads. Incubate overnight on a constant roller at 4°C.
10. Transfer the cell lysate/streptavidin beads to a Wizard^R^Minicolumn. Wash the beads with buffer in the following order: 10 ml of 2% SDS, 10 ml of RIPA buffer, 10 ml of 0.5M NaCl, 10 ml of 2M Urea/50 mM Tris-HCl pH8, and 10 ml of 50 mM ammonium bicarbonate.
11. Resuspend the beads in 120 μl of 50 mM ammonium bicarbonate solution, transfer the beads to a fresh Eppendorf tube.
12. Protein elution for western blot analysis.
  a. Mix 20 μl of beads from Step 11 with 20 μl of 2x Laemmli buffer.
  b. Boil for 5 min at 98°C to elute biotinylated proteins.

**Pause point:** Boiled protein samples in Laemmli buffer can be frozen and stored at −20°C for up to 3 months before proceeding to western blot analysis.

**Figure 4:**
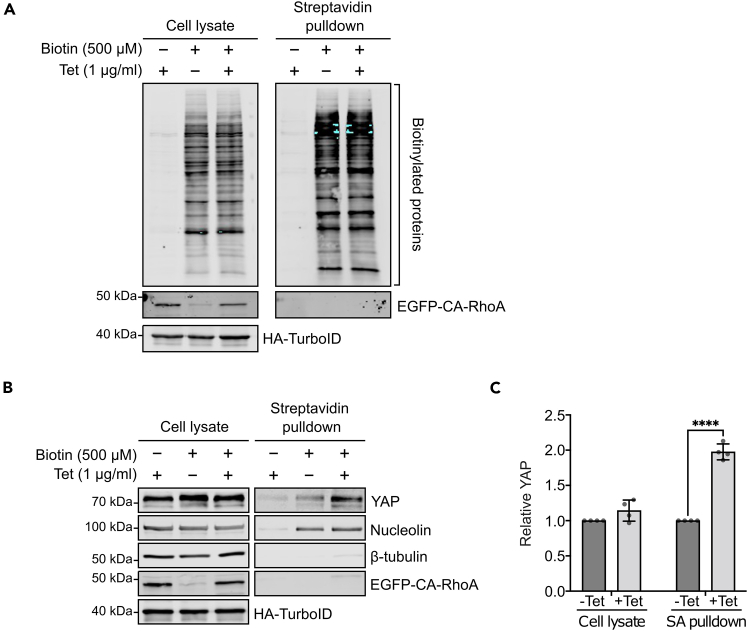
Enrichment and biotinylation of nuclear proteins in HEK293-tet-RhoA-TurboID (A) HEK293-tet-RhoA-TurboID were treated with tetracycline for 2 h followed by biotin for 20 min. The stably expressing TurboID was detected by western blotting with anti-HA antibody. The biotinylated proteins in samples from cell lysate and nuclear fraction (streptavidin pulldown) were analyzed by western blotting with Alexa Fluor™ 680 Streptavidin Conjugate. (B) Western blot of indicated proteins in total cell lysate and nuclear fraction (streptavidin pulldown). Nucleolin and β-tubulin were used as nuclear and cytosolic marker respectively. (C) Quantification shows the normalized YAP expression from B. Replicates=4. Values are means ± s.d. ∗∗∗∗*p* < 0.0001.

13. Western blot analysis.
  a. Detect biotinylated proteins by western blotting using Alexa Fluor™ 680 Streptavidin Conjugate.
  b. Analyze YAP expression in nuclear fraction by standard western blotting. Also blot the samples with nucleolin (nuclear marker) and β-tubulin (cytosolic marker) to confirm the selectivity of the streptavidin pulldown (Figure 4).

### Sample preparation for mass spectrometry

**Timing: 2 days**

This section describes the on-bead reduction, alkylation, and tryptic digestion of biotinylated proteins from Step-11 and the subsequent desalting using C18 stage tips to clean up peptide samples before mass spectrometry analysis.

14. On-bead tryptic digestion.
  a. Wash the remaining cell lysate/streptavidin beads (around 100 μl) with 1 ml of 50 mM ammonium bicarbonate solution by centrifugation at 1,800 x *g* for 3 min at room temperature.
  b. Remove the supernatant. Incubate the beads in 200 μl of 50 mM ammonium bicarbonate containing 10 mM Tris(2-carboxyethyl)phosphine (TCEP) for 15 min at 37°C with shaking.
  c. Add 4 μl of 0.5 M IAA (Iodoacetamide) to alkylate sample for 15 min in the dark at 37°C with shaking.
  d. Add 1 μg of trypsin to digest the sample overnight at 37°C with shaking.
  e. The following day, centrifuge sample at 1,800 x *g* for 1 min at room temperature. Transfer the supernatant (containing the peptide fragments) to a clean Eppendorf tube.
  f. Add 14 μl of 10% Trifluoroacetic acid (TFA) to acidify samples to a pH of 3. Use pH indicator strips to monitor pH changes.

**Pause point:** Acidified peptide samples can be stored at −80°C for up to 6 months before proceeding to the next step. Ensure to seal the samples tightly to prevent evaporation and avoid repeated freeze-thaw cycles.

15. Peptide desalting with Pierce C18 stage tip.
  a. Use a clean pair of scissors to remove approximately 1/4 from the top and bottom of a 1 ml blue tip. The modified tip will connect the C18 stage tip to an uncut 1 ml tip for stable sample loading and washing.
  b. Wash the C18 stage tip with the addition of 100 μl of 0.1% TFA/50% acetonitrile (ACN). Ensure full wetting of the C18 resin for optimal binding.
  c. Transfer the acidified peptides from Step 14f into the prepared C18 stage tip assembly.
  d. Flick gently to remove air bubbles and ensure even resin contact.
  e. Slowly pipette the sample through C18 stage tip.
  f. Collect the flow-through in the original sample tube. Repeat the above steps for improved binding.
  g. Wash the C18 stage tip three times with 100 μl of 0.1% TFA to remove salts and impurities.
  h. Slowly elute peptides with 100 μl of 0.1% TFA/50% ACN, collecting the flow-through in a fresh 2 ml Eppendorf tube.
  i. Dry the purified peptides in a vacuum concentrator for 90 min at 45°C.
  j. Add 10–40 μl of 0.5% formic acid and incubate at 21°C with shaking for 10 minutes. The volume used to resuspend the peptides will depend on the injection volume specified in the LC-MS/MS analysis below.
    **Pause point:** The peptide solution obtained after formic acid incubation can be stored at −80°C for up to 6 months. Ensure to seal the samples tightly to prevent evaporation. Avoid repeated freeze-thaw cycles.
  k. Transfer the peptide solution to an autosampler vial compatible with the liquid chromatography system to be used for LC-MS/MS analysis.
    ***Note:*** C18 spin columns can be used instead of C18 Stage tips.

### Mass spectrometry data acquisition

**Timing: 0.5–2 h per sample, depending on the scan rate of the instrument and the coverage required**

This section describes the proteomic analysis using liquid chromatography tandem mass spectrometry (LC-MS/MS), peptide identification and quantification using MaxQuant and downstream data analysis using Perseus software.

16. Inject 5–10 μl of sample from Step 15j into a nanoflow liquid chromatography tandem mass spectrometry (LC-MS/MS) for peptide analysis.
  ***Note:*** We performed the analysis using an Orbitrap Elite Hybrid Mass Spectrometer (Thermo Fisher Scientific) coupled online to an UltiMate RSLCnano LC System (Dionex). The system was controlled by Xcalibur 3.0.63 (Thermo Fisher) and DCMSLink (Dionex).
  a. Run QC samples to benchmark the system performance e.g., 100 fmol of a BSA digest to check chromatography and MS sensitivity and/or 100 ng of a HeLa digest to assess proteome coverage.
  b. Generate an LC-MS/MS method that includes either a direct injection single column configuration or a two-column configuration which allows for online desalting and separation.
  c. Desalt peptides on-line using an Acclaim PepMap 100 C18 nano/capillary BioLC, 100A nanoViper 20 mm x 75 μm I.D. particle size 3 μm (Fisher Scientific) at a flow rate of 5 μl/min.
  d. Separate peptides using a 125-min gradient from 5 to 35% buffer B (0.5% formic acid in 80% acetonitrile) using an EASY-Spray column, 50 cm × 50 μm ID, PepMap C18, 2 μm particles, 100 Å pore size (Fisher Scientific) at a flow rate of 0.25 μl/min.
  e. Perform DDA LC-MS/MS analysis using an Orbitrap Elite with a cycle of one MS (in the Orbitrap) acquired at a resolution of 60,000 at m/z 400, with the top 20 most abundant multiply charged (2+ and higher) ions in a given chromatographic window subjected to MS/MS fragmentation (CID) in the linear ion trap.
  f. Set an FTMS target value of 1e6 and an ion trap MSn target value of 1e4 with the lock mass (445.120025) enabled to maintain high mass accuracy.
  g. Set the maximum FTMS scan accumulation time to 100 ms and the maximum ion trap MSn scan accumulation time to 50 ms.
  h. Enable dynamic exclusion with a repeat duration of 45 s with an exclusion list of 500 and an exclusion duration of 30.
    ***Note:*** Sample may be analyzed using either data-dependent analysis (DDA) or data-independent analysis (DIA).
    ***Note:*** Newer generations of Orbitrap or Q-TOF instruments will offer a more sensitive and deeper analysis.

### Mass spectrometry data analysis

**Timing: 1 day**

17. Analyze raw MS data.
  a. Analyze raw data with MaxQuant (version 1.6.2.6 or newer) using an up-to-date human UniProt database (UP000005640).
  b. Set the search parameters as follows: digestion set to Trypsin/P with maximum of 2 missed cleavages, methionine oxidation and acetylation at N-terminal peptide as variable modifications, carbamidomethylation at cysteine as fixed modification,
  c. Enable between runs with a match time window of 0.7 min and a 20-min alignment time window, label-free quantification (LFQ) enabled with a minimum ratio count of 2, minimum number of neighbors of 3 and an average number of neighbors of 6.
  d. Set a first search precursor tolerance of 20 ppm and a main search precursor tolerance of 4.5 ppm for FTMS scans and a 0.5 Da tolerance for ITMS scans.
  e. Set a protein false discovery rate (FDR) of 0.01 and a peptide FDR of 0.01 for identification level cut-offs.

***Note:*** Newer versions of MaxQuant are available.

***Note:*** Search tolerances should be set in line with the mass accuracy of the analyzers used for MS1 and MS2 scans.

18. Export MaxQuant proteinGroups output.txt file to Perseus (version 1.5.6.0 or newer) for downstream data analysis.
  a. Load the LFQ Intensities to the main category and the protein and gene names to the Text category.
  b. Group samples by giving replicates of the same group the same name using the Categorical Annotation Rows function.
  c. Log2 transform the LFQ intensity data.
  d. Remove proteins quantified by fewer than three replicates in any one group.
  e. Generate a histogram of the quantitative data to assess the distribution; a normal distribution is needed for the downstream statistical analysis to be valid.
  f. To assess the reproducibility of replicates with groups, calculate the Pearson correlations using the Column correlation function and visualize using the multi-scatter plot function.
  g. Impute missing values using a downshift from the normal distribution, start with the default settings, but some adjustment may be required depending on the dataset.
  h. Perform a PCA analysis to visualize the differences between the groups and their replicates.
  i. To assess the significance of the quantitative differences between groups (e.g., +/− Tet addition to induce Rho), perform a two-sample t-test with a permutation-based FDR of 0.05. The size effect can be given weight by adjusting the S0 parameter, which is typically between 0.1 and 2.
  j. The results of the statistical analysis can be visualized by plotting a volcano plot with the same parameters.

***Note:*** The adjustment of the S0 value will produce sets of significant proteins with varying fold changes, but the overall FDR will be maintained at 0.05. An additional fold-change cutoff may be applied to the data.

### Validation of mechanosensitive nuclear localization of candidate proteins

**Timing: 1 week**

**Figure 5:**
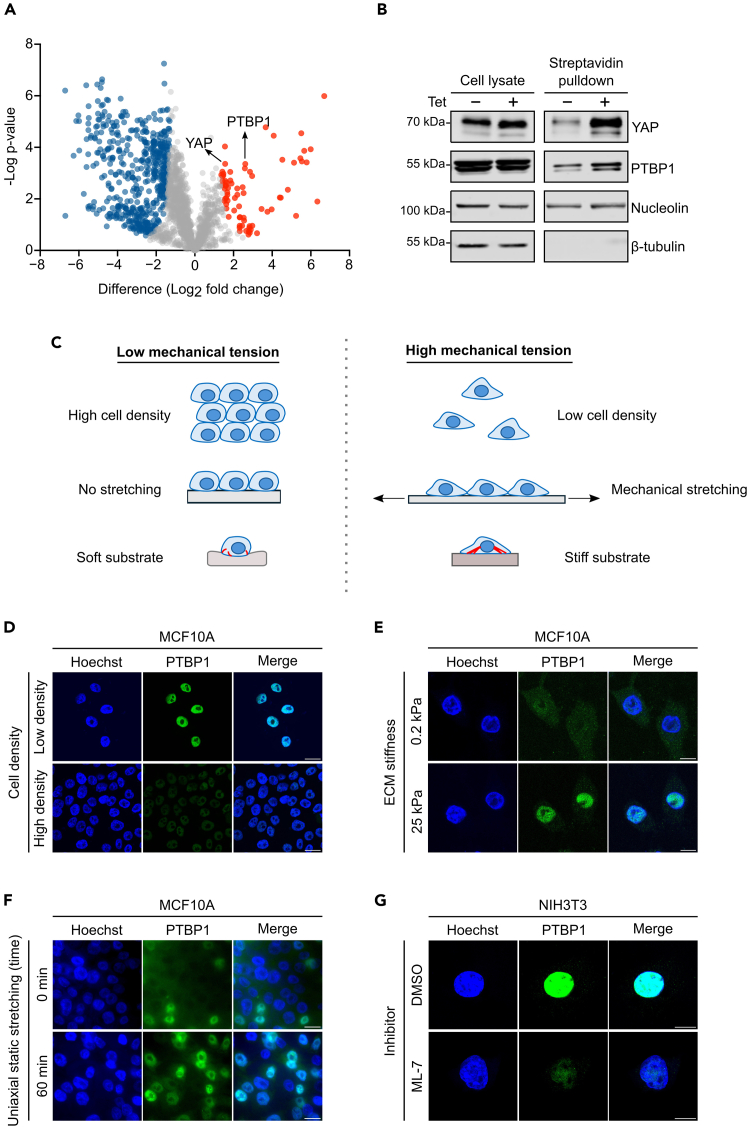
Mechanosensitive responses of PTBP1 under various mechanical conditions (A) Volcano plot demonstrating significant nuclear enrichment of PTBP1 following tetracycline treatment (FDR< 0.01). Reprinted data with permission from Tseng et al.^1^ (B) Western blot analysis of PTBP1 nuclear enrichment, with YAP as a positive control. (C) Diagram shows the mechanical conditions used in this protocol to verify PTBP1 expression. Immunofluorescence microscopy confirming the nuclear accumulation of PTBP1 induced by high mechanical tension including low cell density (D, scale bar: 20 μm), stiff substrate (E, scale bar: 10 μm), and mechanical stretching (F, scale bar: 50 μm). (G) Immunofluorescence images showing PTBP1 nuclear reduction in ML-7 treated NIH3T3. Bar represents 10 μm.

Our screening approach for identifying proteins with mechanosensitve nuclear localization is a multi-layered approach. The first layer is the unbiased identification of proteins that change nuclear levels after controlled expression of constitutively active RhoA. The second layer of our screen is to test the effect of defined mechanical cues more directly on the subcellular localization of candidate proteins identified. The mechanical cues we have tested comprise cell density, extracellular matrix stiffness, and cell stretching. Finally, to demonstrate the direct regulation by actomyosin contractility, inhibitor experiments can be performed. We illustrate our approach using the identified splicing factor PTBP1 as an example (Figure 5).

19. Western blot analysis of nuclear PTBP1 levels in HEK293-tet-RhoA-TurboID.
  a. Plate 5.5x10^5^ HEK293-tet-RhoA-TurboID on 35 mm culture dish.
  b. Culture cells in puromycin-free medium for 24 h, allowing cells to 90% confluency.
  c. Treat the cells with 1 μg/ml tetracycline for 2 h at 37°C followed by labelling of nuclear proteins with 500 μM biotin for 20 min at 37°C.
  d. Aspirate culture medium and wash the cells five times with ice-cold PBS to remove excess biotin.
  e. Prepare cell lysate as Step 4c.
  f. Isolate and collect nuclear fraction using streptavidin pulldown as described in Step 8–12.
  g. Detect PTBP1 protein expression by western blotting using a PTBP1 antibody.

***Note:*** The samples can be frozen at −20°C for future use, or you can directly proceed to western blot analysis.

20. Immunofluorescence imaging of PTBP1 in MCF10A cultured at varying cell densities.
  a. Seed MCF10A at low (0.8x10^5^) or high (5x10^5^) density on Poly-L-Lysine coated glass coverslips in a 12-well plate.
  b. Culture the cells for 24 h under standard condition to reach low cell density (30% confluency) or high cell density (90% confluency).
  c. Perform standard immunofluorescence staining as described in Step 7f-m.
21. Immunofluorescence imaging of PTBP1 in MCF10A cultured on ECM stiffness substrate.
  a. Seed 1x10^5^ MCF10A on soft (0.2 kPa) or stiff (25 kPa) Collagen type I-coated Petrisoft 35 mm dish.
  b. Maintain culture for 3 days under standard condition, ensuring cells remain low cell density (around 30% confluency) at the time of fixation.
  c. Perform standard immunofluorescence staining as described in Step 7f–m.
  d. Analyze images using Olympus Fluoview 1000 Confocal microscopy with 60x water immersion objective.
22. Immunofluorescence staining of PTBP1 under static cell stretching.
  a. Prepare a 100 μg/ml collagen type I coating solution in 1 mM sterile HCl.
  b. Place the PDMS stretch chamber in a 100 mm petri dish.
  c. Add 1 ml collagen solution per well, ensuring full surface coverage.
  d. Cover the petri dish with a lid and incubate the chamber at 37°C for 4 h.
  e. Aspirate the collagen coating solution, rinse the chamber twice with serum-free culture medium.
  f. Seed 5x10^5^ MCF10A cells per well on the coated chamber and culture for 24 h under standard condition. The cells should reach approximately 90% confluency before stretching.
  g. Mount the chamber on the STREX stretch device. Apply 10 % static stretch and maintain at 37°C for 1 h.
  h. Fix the cells with PFA fixation buffer as described in Step 7f.
  i. Aspirate PFA fixation buffer. Carefully cut out the PDMS membrane with a clean scalpel and transfer the membrane with cells up to a 35 mm dish.
  j. Perform standard immunofluorescence staining with PTBP1 primary antibody as described in Step 7g-m.

***Note:*** We perform uniaxial cell stretching using a manual stretch device (STREX, ST-0040) according to manufacturer’s instruction (https://strexcell.com/cell-stretching-system/#STB-100). In this protocol, we culture cells on the 4-well PDMS uniaxial stretch chamber (2 cm^2^ per well).

23. Immunofluorescence analysis of PTBP1 in myosin light chain kinase inhibitor ML-7 experiments.
  a. Seed 1x10^5^ NIH3T3 cells on Poly-L-Lysine-coated glass coverslips in 12-well dish.
  b. Maintain the cells for 24 h under standard condition.
  c. Treat cells with 80 μM ML-7 or 0.1% DMSO (control) at 37°C for 30 min.
    ***Note:*** Effective ML-7 concentration might vary between cell types. We recommend using YAP localization as a positive control to optimize concentration and incubation time. The optimal concentration is the minimum that effectively excludes YAP from the nucleus while maintaining overall cell integrity.
  d. Perform standard immunofluorescence staining with PTBP1 primary antibody as described in Step 7f-m.

## Expected outcomes

The stable HEK293-tet-RhoA-TurboID cell line was established to modulate actomyosin contractility via controlled expression of constitutive active RhoA. This approach can also be adopted for cell lines of a different origin. If stable cell lines are successfully established, cells are expected to express constitutively active RhoA variant (CA-RhoA) under the control of a tetracycline inducible promoter allowing control over time and expression levels of the EGFP tagged CA-RhoA, which can be easily monitored and quantified by western blotting (Figure 2A). In addition, TurboID mediated protein biotinylation is expected to occur and can be detected using Alexa Fluor™ 680 Streptavidin Conjugate (Figure 2B). Furthermore, tetracycline induced CA-RhoA signalling is expected to trigger actomyosin contractility, which can be validated using traction force microscopy (Figure 3A). To further confirm mechanosensitive nuclear localization of candidate proteins, the tetracycline induced CA-RhoA signaling should override inhibitory effect of high cell density on YAP nuclear localization with the consequence of an increase in nuclear YAP after tetracycline treatment (Figure 3B). The NLS-tagged TurboID is expected to be targeted to the nucleus to promote exclusive biotinylation of nuclear proteins in the presence of exogenous biotin, biotinylation can be monitored via western-blotting using conjugated streptavidin (Figure 4A). To confirm the specificity of the labelling for nuclear proteins, the streptavidin pulldown fraction should contain the nuclear marker protein nucleolin but should be devoid of cytosolic marker proteins such as β-tubulin. YAP should be included as a positive control and should be enriched in the pulldown samples treated with tetracycline compared to mock treated samples (Figures 4B and 4C).

For the downstream proteomics analysis, we applied a threshold of a 2-fold change. This analysis identified 74 proteins enriched in the nucleus after tetracycline treatment. YAP served as a positive control and should be found among the enriched protein hits to validate the screen approach (Figure 5A). The results from the proteomic approach should be further validated via western blotting (Figure 5B). To confirm that the nuclear accumulation of candidate proteins is due to changes in actomyosin contractility, further experiments are necessary. Proteins are expected to demonstrate changes in nuclear accumulation in response to ML-7 inhibitor treatment affecting actomyosin contractility or in response to mechanical cues known to affect actomyosin contractility such as cell density, cell size, ECM stiffness or cell stretching^20^^,^^21^^,^^22^^,^^23^ (Figures 5C–5G).

## Limitations

Our screening approach assumes that mechanical forces applied to the nucleus alter nucleocytoplasmic shuttling of proteins by affecting nuclear pore permeability and transport dynamics. However, given the two-hour timescale of tetracycline treatment, we cannot entirely exclude the possibility that some observed changes in nuclear protein levels result from rapid transcription and translation, reflecting mechanosensitive gene expression. Importantly, this limitation does not undermine the potential involvement of these candidate proteins in a mechanotransduction pathway.

Furthermore, RhoA/ROCK signaling can also be activated by mechanical cue-independent pathways, primarily through receptor signaling, and as part of cellular stress responses.^24^^,^^25^^,^^26^ Thus, several of the nuclear enriched proteins found in the mass spectrometry screen might reflect mechanical cue independent RhoA/ROCK signaling and follow up validation using more direct mechanical cues as described in our protocol above is essential.^25^^,^^27^

## Troubleshooting

### Problem 1

Unhealthy cells caused by excessive CA-RhoA mediated actomyosin contractility.

### Potential solution

Optimize both tetracycline dose and induction time. Monitor CA-RhoA expression by western blotting as described in Step 4. Evaluate cell morphology of each tetracycline treatment condition using a microscope. Select the optimal concentration and induction duration that promote YAP nuclear enrichment while maintaining normal cell viability and morphology, as described in Step 7.

### Problem 2

**Figure 6:**
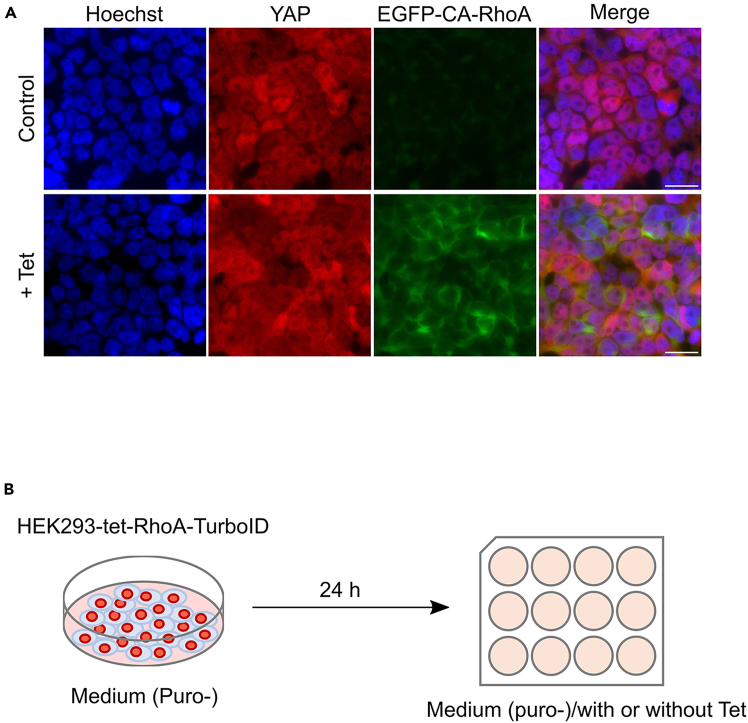
Persistent nuclear localization of YAP in HEK293-tet-RhoA-TurboID (A) Immunofluorescence images showing nuclear accumulation of YAP in HEK293-tet-RhoA-TurboID, independent of tetracycline treatment. Scale bar: 20 μm. (B) Schematic diagram illustrating the preculture of cells in puromycin-free medium for 24 h prior to induction of EGFP-CA-RhoA expression by tetracycline.

Unexpected nuclear YAP caused by the selection antibiotic Puromycin (Figure 6A).

### Potential solution

Only use medium supplemented with puromycin during standard cell culture to maintain genetic integrity of the stable HEK293-tet-RhoA-TurboID line. Replenish culture medium without puromycin at least 24 h prior to tetracycline treatment and biotin labelling, as described in Step 7d-e and 19b-c (Figure 6B).

### Problem 3

Heterogenous biotinylation during proximity labelling in high-density culture, may reduce nuclear proteome coverage and induce background noise.

### Potential solution

Optimize both biotin concentration and treatment duration to balance labelling efficiency while minimizing the background. In this protocol, we treat cells with 500 μM biotin for 20 min as described in Step 8c. After labelling, thoroughly wash cells five times with ice-cold PBS to remove unincorporated biotin, as described in Step 8d. Use proper amounts of streptavidin agarose beads to recover biotinylated proteins while minimize non-specific binding (see Step 8f). For sample preparation for mass spectrometry analysis, include YAP as positive control for all streptavidin pulldown experiments to minimize batch-to-batch variation (see Step 13).

## Resource availability

### Lead contact

Further information and requests of resources and reagents should be directed to the lead contact, Kai S. Erdmann (k.erdmann@sheffield.ac.uk).

### Technical contact

Technical questions regarding the execution of this protocol should be directed to and will be answered by the technical contact, Pei-Li Tseng (p.tseng@sheffield.ac.uk).

### Materials availability

This study did not generate new unique reagents.

### Data and code availability

Proteomic data have been published and deposited in the ProteomeXchang (PRIDE) under the accession number PRIDE: PXD046157 (Tseng et al.^1^). No custom code was generated in this study.

## Acknowledgments

The plasmid pcDNA3-EGFP-RhoA-Q63L was a gift from Gary Bokoch (Addgene plasmid # 12968), and 3xHA-TurboID-NLS_pCDNA3 was a gift from Alice Ting (Addgene plasmid # 107171). We thank Annica K. B. Gad, Ahmed Salem, and Sarah C Macfarlane for traction force microscopy measurements. We also like to thank the Wolfson Light Microscopy Facility, University of Sheffield, for their expert assistance with Olympus FV1000 confocal imaging.

This work was supported by a fee scholarship and a publication scholarship from the University of Sheffield awarded to W.S.

## Author contributions

All authors contributed to the writing and editing of the manuscript. P.-L.T. and W.S. performed the unpublished experiments. M.O.C. and P.-L.T. performed the mass spectrometry analysis. K.S.E., M.O.C., and P.-L.T. conceptualized the screening approach.

## Declaration of interests

The authors declare no competing interests.
